# Effects of Pharmaceuticals and Endocrine-Disrupting Chemicals on Reproductive Biology of Aquatic Fauna: Penguins as Sentinel Species

**DOI:** 10.3390/jox15040110

**Published:** 2025-07-04

**Authors:** Grace Emily Okuthe, Edith Dube, Patrick Siyambulela Mafunda

**Affiliations:** 1 Department of Biological & Environmental Sciences, Faculty of Natural Sciences, Walter Sisulu University, Mthatha 5117, South Africa; edube@wsu.ac.za; 2 Department of Human Biology, Faculty of Medicine and Health Science, Walter Sisulu University, Mthatha 5117, South Africa; pmafunda@wsu.ac.za

**Keywords:** aquatic ecosystems, aquatic fauna, endocrine-disrupting chemicals, penguins, pharmaceuticals, pollution, reproductive physiology

## Abstract

The escalating global contamination of aquatic ecosystems by pharmaceuticals and endocrine-disrupting chemicals (EDCs) stemming from diverse anthropogenic sources represents a critical and pervasive threat to planetary Earth. These contaminants exhibit bioaccumulative properties in long-lived organisms and undergo trophic biomagnification, leading to elevated concentrations in apex predators. This review synthesizes current knowledge regarding the far-reaching impacts of pharmaceutical and EDC pollution on the reproductive biology of aquatic fauna, focusing on the heightened vulnerability of the endangered African penguin. A rigorous literature review across key scientific databases—PubMed, Scopus, Web of Science, and Google Scholar—using targeted search terms (e.g., penguins, contaminants of emerging concern, penguin species, seabird species, Antarctica, pharmaceuticals, personal care products, EDCs) underpins this analysis. This review explores the anthropogenic sources of pharmaceuticals and EDCs in aquatic ecosystems. It discusses the mechanisms by which these chemicals disrupt the reproductive physiology of aquatic fauna. Recent studies on the ecological and population-level consequences of these contaminants are also reviewed. Furthermore, the review elaborates on the urgent need for comprehensive mitigating strategies to address their effects on vulnerable penguin populations. These approaches hold the potential to unlock innovative pathways for conservation initiatives and the formulation of robust environmental management policies aimed at safeguarding aquatic ecosystems and the diverse life they support.

## 1. Introduction

**Figure 1:**
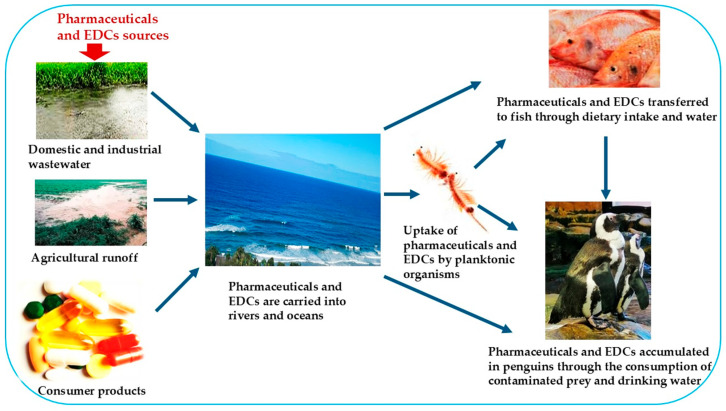
Pathways of pharmaceutical and endocrine-disrupting chemical contamination in aquatic ecosystems.

Aquatic ecosystems are increasingly exposed to complex mixtures of anthropogenic pollutants, among which pharmaceuticals and endocrine-disrupting chemicals (EDCs) pose growing ecological and health concerns. Originating from human and veterinary medicine, agriculture, industrial processes, and domestic products, these contaminants enter water bodies through wastewater effluent, agricultural runoff, and leaching from consumer goods [1,2]. Due to their bioactive nature and persistence, pharmaceuticals and EDCs accumulate in aquatic organisms and can interfere with hormonal regulation, making reproductive toxicity one of their most critical impacts [3,4]. Figure 1 illustrates the pathways of pharmaceuticals and EDC contamination in aquatic ecosystems.

Pharmaceuticals, including over-the-counter drugs, antibiotics, and personal care products, are increasingly recognized for their unintended endocrine activity [5]. EDCs, such as bisphenol A, phthalates, dioxins, and atrazine, alter hormonal pathways by mimicking, blocking, or modifying natural hormone signals [6]. These substances have been detected in surface waters globally, as confirmed by recent studies and shown in Table 1 and Table 2. However, chemical concentrations in surface water often differ significantly from those found in aquatic organisms due to processes such as bioaccumulation and biomagnification [7,8]. Lipophilic EDCs tend to accumulate in the fatty tissues of aquatic species, leading to internal concentrations that may exceed ambient water levels to some extent. For example, studies have reported internal concentrations of bisphenol A and phthalates in fish and marine mammal tissues, reaching levels that are associated with adverse reproductive effects, even when surface water concentrations appear low [9,10]. This bioaccumulative potential is especially concerning for long-lived species and top predators, where EDCs can magnify across trophic levels. Internal exposure to these contaminants has been linked to impaired gametogenesis, reduced fertility, and disrupted sexual development across a range of aquatic taxa [11].

Although numerous reviews have addressed the effects of EDCs on aquatic environments, this review offers a distinct contribution by also focusing specifically on pharmaceuticals as emerging reproductive toxicants and by evaluating their impacts across a wide taxonomic range, including invertebrates, fish, marine mammals, and seabirds. Furthermore, it highlights the underexplored role of penguins as sentinel species, offering insights into pollutant exposure and reproductive disruption in remote marine environments.

Penguins, due to their long lifespan, high trophic level, and dependence on marine food webs, are particularly vulnerable to bioaccumulation and biomagnification of pharmaceuticals and EDCs. Studies have detected persistent pollutants such as PCBs, PBDEs, PFAS, and pharmaceutical residues in penguin tissues, feathers, and eggs, even in remote Antarctic regions [27,28]. Their site fidelity and accessible nesting colonies make them ideal indicators for long-term environmental monitoring [29]. By examining reproductive effects in penguins and other sentinel species, this review offers a comprehensive synthesis of contamination trends, biological impacts, and mechanisms of action. It also identifies knowledge gaps and proposes future directions for research, conservation, and policy development aimed at mitigating the reproductive risks posed by pharmaceutical and EDC contamination in marine ecosystems.

## 2. Methodology of the Review

This review was conducted through a literature search using scientific databases: PubMed, Scopus, Web of Science, and Google Scholar. The search strategy incorporated specific keywords and phrases, such as penguins, emerging contaminants, pharmaceuticals, endocrine-disrupting chemicals, marine pollution, reproductive toxicity, and sentinel species. Only peer-reviewed articles, reports, and reviews published mostly between 2010 and 2025 were considered. Inclusion criteria required studies to directly assess or provide relevant data on the impacts of pharmaceuticals or EDCs on aquatic fauna, with a particular focus on marine birds and mammalian species. Studies were excluded to ensure relevance and quality. Criteria included: limited focus on reproductive biology or lacking direct relevance to pharmaceuticals, endocrine-disrupting chemicals, or their impacts on aquatic life, particularly marine birds and mammals, non-English language, and duplicate entries. The search initially yielded 17,405 articles. Titles and abstracts were screened for relevance to the review scope, narrowing the selection to 180 full-text articles. These were thoroughly analyzed to extract detailed information aligned with the review’s core themes. The information collected was organized into a structured review, ensuring clarity, coherence, and an emphasis on high-quality journal sources.

## 3. Pharmaceuticals and EDCs in Aquatic Environments

Pharmaceuticals and endocrine-disrupting chemicals (EDCs) enter aquatic environments through multiple sources and pathways, posing significant risks to aquatic organisms and ecosystems. Understanding these sources and transport mechanisms is crucial for developing effective mitigation strategies.

### 3.1. Sources of Pharmaceuticals and EDCs

**Figure 2:**
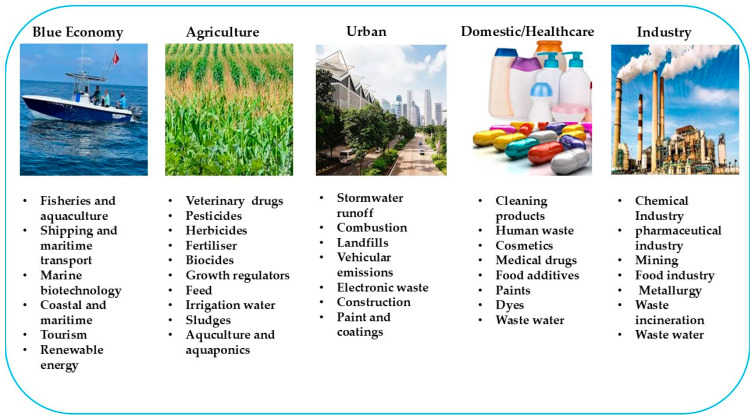
Sources of pharmaceuticals and endocrine-disrupting chemicals (EDCs) in qquatic environments.

Pharmaceuticals and EDCs enter aquatic environments through various pathways, presenting significant risks to ecosystems, wildlife, and human health [4,11]. Figure 2 shows the sources of pharmaceuticals and EDCs in aquatic environments.

#### 3.1.1. Domestic and Municipal Wastewater as Sources of Pharmaceuticals and EDCs

Domestic and municipal wastewater is a key source of pharmaceuticals and EDCs in aquatic environments. For example, 15 priority pollutants were identified in Chinese wastewater, including PFAS, PCBs, pesticides, pharmaceuticals, and endocrine disruptors, highlighting their persistence [30]. A major contributor is human excretion, as many pharmaceuticals are only partially metabolized and enter sewage systems through urine and feces. Personal care products like cosmetics, detergents, and fragrances also release EDCs when washed off during bathing and cleaning [31]. Improper disposal, such as flushing medications or discarding them in landfills, further contaminates water sources. Hospital and laboratory waste also adds to the pollution, introducing pharmaceutical residues and chemical contaminants into wastewater systems [32].

In wastewater treatment plants, incomplete removal of these contaminants during treatment allows them to persist in effluents, which are then discharged into rivers, lakes, and coastal waters [33]. Studies have detected pharmaceuticals in wastewater at concentrations from nanograms to micrograms per liter [34,35,36,37]. Surface water pharmaceuticals ranging from 0.44 ng/L for ciprofloxacin to 19,000 ng/L for metformin have been reported [38]. Conventional wastewater treatment plants cannot fully remove these pollutants, allowing them to persist in treated effluents. Continuous discharge into rivers, lakes, and coastal waters threatens aquatic life and leads to bioaccumulation and biomagnification.

#### 3.1.2. Industrial Effluents as Sources of Pharmaceuticals and EDCs

Industrial effluents are also a major source of pharmaceuticals and EDCs in aquatic ecosystems. These pollutants originate from various industries, either as raw materials or by-products, and persist in the environment due to ineffective wastewater treatment. Pharmaceutical manufacturing facilities release active pharmaceutical ingredients, including antibiotics and hormones, into wastewater, contributing to contamination [32]. A broad spectrum of antibiotics, including ciprofloxacin, ofloxacin, enrofloxacin, orbifloxacin, azithromycin, clarithromycin, sulfapyridine, sulfamethoxazole, trimethoprim, nalidixic acid, pipemidic acid, oxolinic acid, cefalexin, clindamycin, metronidazole, ampicillin, and tetracycline was identified in the final effluents of wastewater treatment plants across seven European countries (Portugal, Spain, Ireland, Cyprus, Germany, Finland, and Norway) [39]. Similarly, high concentrations of pharmaceuticals, particularly sulfamethoxazole, were reported in untreated wastewater from treatment plants in South Africa’s Eastern Cape province [40]. These findings highlight the widespread presence of pharmaceutical contaminants in treated and untreated effluents, raising concerns about their persistence in aquatic ecosystems and potential environmental and public health risks.

Beyond pharmaceuticals, the chemical and plastic industries significantly contribute to EDC pollution [41]. Common industrial chemicals such as bisphenol A (BPA) and phthalates leach into water sources, disrupting endocrine functions in both wildlife and humans. BPA leaching of up to 42.78 ppm has been reported from food-grade plastics, with black poly bags, sliced juice bottles, and infant milk bottles containing detectable levels [42]. The leaching of EDCs from plastics indicates that industrial plastic production and disposal contribute to EDC contamination in water systems. Plastic can degrade into microplastics. Microplastics serve as effective carriers of EDCs due to their high surface-area-to-volume ratio and hydrophobic nature, which enhances their capacity to adsorb persistent organic pollutants such as bisphenols, phthalates, and alkylphenols from the surrounding environment. These contaminants can accumulate on the surface of microplastics during their transport through aquatic systems, effectively concentrating EDCs from diluted sources [43].

When microplastics are ingested by aquatic organisms, the adsorbed EDCs may desorb in the gastrointestinal tract under conditions such as changes in pH and enzymatic activity. This desorption increases the bioavailability of EDCs, allowing them to cross biological membranes and enter systemic circulation, potentially leading to bioaccumulation and adverse physiological effects [44]. Moreover, as contaminated prey organisms are consumed by predators, EDCs can be transferred through the food web, thereby amplifying exposure at higher trophic levels. This trophic transfer poses a significant ecological threat, particularly to species at the top of the food chain, including fish, birds, and marine mammals, and may ultimately impact human health through seafood consumption [45].

The textile and paper industries also discharge EDCs, particularly per- and polyfluoroalkyl substances (PFAS), which are widely used in water and stain-resistant coatings. PFAS are highly persistent in the environment, bioaccumulate in organisms, and are linked to endocrine disruption, immune system impairments, and other adverse health effects [46]. Their widespread presence in industrial effluent shows the urgent need for regulatory interventions.

#### 3.1.3. Agricultural Runoff as a Source of Pharmaceuticals and EDCs

Agricultural activities contribute significantly to contaminating aquatic ecosystems with pharmaceuticals and EDCs. These pollutants enter water bodies through surface runoff, leaching, and improper waste disposal, posing risks to both environmental and human health [47]. The major sources of contamination from agricultural runoff include veterinary pharmaceuticals, pesticides, and aquaculture practices.

Livestock farming extensively uses antibiotics, hormones, and antiparasitic drugs to enhance growth, prevent infections, and treat diseases. However, these compounds are excreted in animal waste [48,49,50] and can enter water bodies if not properly managed through manure runoff. High concentrations of veterinary antibiotics were detected in animal manure and manure-based fertilizers from Jiangsu Province, China, with sulfonamides, fluoroquinolones, and tetracyclines being prevalent. Ciprofloxacin and chlorotetracycline were commonly found in manure, while tetracycline dominated fertilizers, reaching up to 1920 mg/kg [51]. Similarly, sulfonamides, tetracyclines, quinolones, macrolides, amphenicols, and hormones such as 17β-estradiol and estrone were identified [49]. These pharmaceuticals persist in the environment, potentially harming aquatic life. Additionally, growth hormones like estradiol and trenbolone interfere with endocrine functions in aquatic organisms, leading to reproductive and developmental abnormalities.

Many agrochemicals used in crop protection possess endocrine-disrupting properties and can infiltrate water systems through runoff and leaching. Regions with intensive agricultural activity are particularly vulnerable to aquatic pesticide and herbicide pollution. The presence of herbicides, including atrazine, metolachlor, simazine, and terbuthylazine, was reported in various water sources within the Mangaung Metropolitan Municipality, South Africa [52]. In a different study, sixty pesticide compounds were identified in three South African river catchments, with all samples containing at least three pesticides and 83% having five or more. Among these, terbuthylazine, imidacloprid, and metsulfuron-methyl were the most concentrated, frequently surpassing environmental quality standards [53]. Such widespread contamination raises significant environmental and human health concerns, as prolonged exposure to these chemicals can disrupt endocrine function and impair ecosystem stability. For example, atrazine is known to interfere with hormone regulation, leading to reproductive abnormalities [54]. Even at trace concentrations, these chemicals interfere with endocrine signalling pathways, leading to developmental, reproductive, and immune system disorders in wildlife and humans [55].

#### 3.1.4. Aquaculture Practices as a Source of Pharmaceuticals and EDCs

Intensive fish farming heavily depends on antibiotics, antifungals, and growth enhancers to maintain stock health and optimize production. To prevent and treat infections, substantial amounts of veterinary antibiotics are either directly administered or mixed into fish feed, with a considerable proportion remaining unmetabolized and accumulating in aquaculture effluents [56]. Common antibiotics found in aquaculture wastewater include oxytetracycline, florfenicol, quinolones, and sulfonamides, often combined with trimethoprim.

EDCs, pharmaceuticals, and personal care products (PPCPs) were identified as key contaminants, with varying concentrations, by fishing seasons in two aquatic product processing sewage treatment plants along the southeast coast of China. The levels of these contaminants followed this trend: EDCs (1877.85–15,398.02 ng/L in influent, 3.37–44.47 ng/L in effluent) > sulfonamide antibiotics (75.14–906.19 ng/L in influent, 1.14–15.33 ng/L in effluent) > PPCPs (44.47–589.93 ng/L in influent, 2.54–34.16 ng/L in effluent) ≈ fluoroquinolones (54.76–434.83 ng/L in influent, 10.75–32.82 ng/L in effluent) > other antibiotics (16.21–51.96 ng/L in influent, 0.68–6.17 ng/L in effluent) [57]. The persistence of these contaminants in effluent poses significant environmental risks. The discharge of medicated feeds and untreated wastewater contaminates surrounding water bodies, contributing to pharmaceutical pollution in aquatic systems. Additionally, synthetic steroids and hormone-mimicking compounds used for enhancing fish growth can disrupt aquatic organisms’ reproductive cycles, leading to ecological imbalances [58].

#### 3.1.5. Landfill and Leachate Runoff as a Source of Pharmaceuticals and EDCs

Pharmaceuticals and EDCs disposed of in landfills pose significant environmental concerns [59]. Landfill leachates from 318 sites globally were analyzed, revealing high concentrations of EDCs with significant variation across countries. Bisphenol A was the most abundant pollutant, frequently detected in the US (0.63–6.38 × 10^3^ μg/L), China (0.98–3.27 × 10^3^ μg/L), and other countries, primarily due to synthetic polymers and thermal paper in landfills. Leachates from China also showed elevated levels of plasticizers, such as di-ethyl phthalate and dimethyl phthalate. Pharmaceuticals and personal care products were abundant in leachates, with notable concentrations of nicotine, caffeine, acetaminophen, and ibuprofen detected in different countries [60]. Similarly, regional variations in pharmaceutical concentrations were observed across China, with certain pharmaceuticals consistently detected in all samples (100% detection). These included diclofenac, sulpiride, gliclazide, sulfadiazine, sulfamethazine, erythromycin-H_2_O, and lincomycin [61]. Unfortunately, these contaminants can leach into groundwater or surface water through percolation, contaminating vital water resources. This contamination may impact human health and aquatic ecosystems, as these substances are not easily broken down and can persist in the environment [59,62].

### 3.2. Pathways of Pharmaceuticals and EDCs in Aquatic Environments

As effluents flow through rivers and streams, pharmaceutical residues and EDCs are transported from urban and agricultural areas to larger bodies of water, like estuaries and oceans, where they can accumulate and cause further environmental damage [63].

Leaching from landfills, septic systems, and agricultural fields enables these contaminants to seep into groundwater through percolation or flow as surface water into larger aquatic systems [59,62]. In aquatic systems, pharmaceuticals and EDCs can enter aquatic organisms through various routes, including waterborne exposure via gills, ingestion through contaminated water or food, and skin absorption. Once inside the organism, these chemicals are transported to various tissues, including target organs such as the liver and kidneys, where they can exert toxic effects or interfere with hormonal systems, particularly EDCs [3]. Invertebrates and fish can accumulate pharmaceuticals and EDCs from contaminated water or sediment, and these substances can biomagnify through the food chain. Pharmaceuticals and EDCs can bioaccumulate in long-lived species such as fish, shellfish, and marine mammals. For instance, predatory fish may accumulate higher concentrations of chemicals by consuming smaller contaminated prey. As predators consume these organisms, contaminants biomagnify, leading to higher concentrations in top predators, including humans who rely on seafood. This trophic transfer through the food web amplifies the risks to human health and biodiversity [63,64]. Pharmaceuticals such as hydrochlorothiazide, gemfibrozil, and ibuprofen, commonly detected in water, were found in a Mediterranean river, while methylparaben was abundant in biofilm. Additionally, ibuprofen was found in Hydropsyche, a type of aquatic insect, highlighting the potential for pharmaceuticals to bioaccumulate and persist in aquatic ecosystems, further emphasizing the risks associated with their presence in the environment [64].

Hydrophobic EDCs, like polychlorinated biphenyls (PCBs) and certain pharmaceuticals, can bind to organic matter in sediments, persisting for long periods. PCB contamination was reported in surface sediments from shrimp ponds in four regions along the northern Central Java coast [65], raising concerns about the impact of PCB buildup on the aquatic ecosystem and local communities reliant on these areas for food and livelihood. Through resuspension, these sediment-bound pollutants can re-enter the water column, where they are redistributed, affecting aquatic organisms. Such contaminants pose long-term environmental risks that persist even after initial contamination [66,67].

Lastly, oceanic currents facilitate the long-range transport of contaminants, carrying pollutants far from their original sources. This global distribution of contaminants disrupts marine ecosystems across vast distances, impacting even remote regions [68,69]. For instance, seventeen organoamine pesticides were detected in the Arctic Ocean, with cycloate, isoprocarb, cyflufenamid, diphenylamine, benalaxyl, tebufenpyrad, pentachloroaniline, and metolachlor being the most abundant [70]. Some of these compounds, like cycloate, isoprocarb, and metolachlor, have raised concerns due to their potential endocrine-disrupting effects, which could harm both aquatic ecosystems and human health. This transboundary pollution shows the complexity of managing pharmaceutical and EDC contamination, highlighting the need for international cooperation in addressing these pervasive environmental threats.

## 4. Reproductive Strategies of Aquatic Fauna

Reproduction, a fundamental biological process, exhibits remarkable diversity across the animal kingdom [71,72], wherein sexual reproduction is the norm [73]. Reproduction in aquatic ecosystems is marked by considerable diversity. Gonochorism, the separation of sexes, is a common reproductive strategy in vertebrates, with teleost fish demonstrating the greatest range of variations, especially in marine habitats [74]. Sexual reproduction requires the syngamy of morphologically distinct gametes, a phenomenon designated as anisogamy [75,76]. Despite the general trend of male gametes being smaller than female gametes [77], sex determination mechanisms display substantial variation. While mammals and birds possess stable sex determination, numerous marine species, notably fish, exhibit variable sex determination shaped by genetic, environmental, and epigenetic factors [78]. Consequently, defining sex by reproductive strategy often proves more informative than relying on physiological characteristics [79].

Gonochorism, also known as dioecy, describes a sexual system where individuals have one of at least two distinct sexes, male or female [80]. Simultaneous hermaphroditism, on the other hand, is the capacity of an individual to reproduce using either male or female gametes within its lifetime. Sequential hermaphroditism is another form of reproductive strategy from gonochorism, although there is some overlap. It may be unclear whether a species is gonochoristic or sequentially hermaphroditic [81,82].

Sex-changing species exhibit sequential hermaphroditism, categorized as protandry when individuals transition from male to female [83] and exemplified by the clownfish (*Amphiprion ocellaris*) and protogyny when the transition is from female to male [84], as seen in zebrafish (*Danio rerio*). The rock wrasse (*Halichoeres semicinctus*; [85] demonstrates protogyny, commencing life with exclusive female gamete production and potentially undergoing sex reversal to exclusively produce male gametes. While superficially resembling gonochorism due to the apparent presence of distinct sexes, these sequentially hermaphroditic taxa are different because individuals can alter their reproductive capacity. A more functional definition of male and female sexes emphasizes their distinct reproductive strategies [79], stemming from the asymmetry of gametes and leading to associated morphological and behavioral sex differences. Species such as *H. semicinctus* are thus classic examples of sequential hermaphroditism [85].

Aquatic fauna also exhibit a range of strategies to ensure the survival of their offspring. While both groups share the commonality of aquatic habitat, significant differences in their reproductive strategies have evolved due to their unique physiological adaptations and ecological niches. Nonetheless, it can be influenced by pharmaceuticals and EDCs. Aquatic fauna, from fish to marine mammals, have developed diverse reproductive strategies. One common approach is external fertilization (Table 3), where eggs and sperm are released into the water column, allowing fertilization to occur in the open environment [86]. This strategy is particularly prevalent among bony fish (Teleostei) and frogs (Anura), with many species engaging in spawning behaviours, releasing large quantities of gametes simultaneously [87]. For example, in certain fish, the males meticulously match the spatial pattern of their sperm to meet the spatial distribution of the eggs [88]. Internal fertilization (Table 3) is another strategy some aquatic animals employ, including certain sharks [89,90,91,92,93], rays, and some fish species and penguins [85,94]. In this case, the male transfers sperm directly into the female’s reproductive tract, increasing the chances of fertilization and providing a more protected environment for embryo development.

Furthermore, aquatic fauna exhibits a range of reproductive modes, including oviparity, ovoviviparity, and viviparity. Like most fish and amphibians, oviparous species lay eggs that develop and hatch outside the mother’s body. As stated above, ovoviviparous species, like some sharks and rays, retain fertilized eggs within the female’s body until they hatch, providing protection. Viviparous species, such as some sharks and marine mammals, nourish their developing young through a placenta-like structure, ensuring a direct supply of nutrients. Aquatic mammals, such as whales, dolphins, seals, and manatees, have evolved unique reproductive strategies to thrive in aquatic environments [95,96,97,98]. Despite their aquatic lifestyle, they retain many mammalian characteristics, including internal fertilization and live birth.

Mating in aquatic fauna also involves complex courtship behaviors and precise timing. This is common for animals living in environments with regular seasonal cycles. The gestation period also varies among species, ranging from a few months in smaller species to over a year in larger whales [99,100], as illustrated in Table 3 and Table 4. These mammals also exhibit a range of maternal care strategies. Some species, such as whales and dolphins, provide extensive care to their young, nursing them for extended periods and teaching them essential survival skills [101]. Others, like seals, may provide less intensive care but rely on their offspring’s innate survival abilities. While aquatic animals often produce large numbers of offspring with relatively little parental care, relying on abundant resources in aquatic environments, aquatic mammals typically produce fewer offspring but invest heavily in their care, ensuring survival in a more challenging and constantly changing environment. Despite divergent reproductive strategies and modes in aquatic fauna, from the gamete morphology perspective, male gametes are usually smaller, and female ones are larger [77].

### 4.1. Aquatic Fauna Reproduction Physiology Disrupted by Pharmaceuticals and EDCs

Environmental endocrine-disrupting chemicals (EDCs) pose a significant threat to aquatic ecosystems by impairing the reproductive success of their fauna [102]. These anthropogenic chemicals interfere with the endocrine systems of aquatic organisms, directly disrupting hormonal regulation, gamete development and function, sexual differentiation, reproductive behavior, and fertilization. Specifically, EDCs can alter sex hormone levels and the hypothalamic–pituitary–gonadal (HPG) axis, leading to reduced sperm count and viability, decreased egg quality, abnormal sperm morphology, sex reversal, intersex conditions, abnormal organ development, and altered mating behaviors [103]. Consequently, these direct impacts result in reduced fertility, lowered reproductive output, and increased reproductive failure, threatening population persistence.

Beyond direct effects, EDCs also exert indirect impacts. They can suppress the immune system [104], disrupt metabolism [105], affect energy allocation for reproduction, and cause developmental abnormalities that later compromise reproductive capacity.

Evidence highlights these detrimental effects. For instance, estrogenic EDCs induce vitellogenin production in male fish, and tributyltin (TBT) causes imposex and sterility in female marine snails. Furthermore, EDCs can skew sex ratios in aquatic populations. EDS significantly and multifacetedly disrupts aquatic fauna reproduction, impacting individual fitness and population sustainability. These are further discussed below:

#### 4.1.1. Gametogenesis

**Figure 3:**
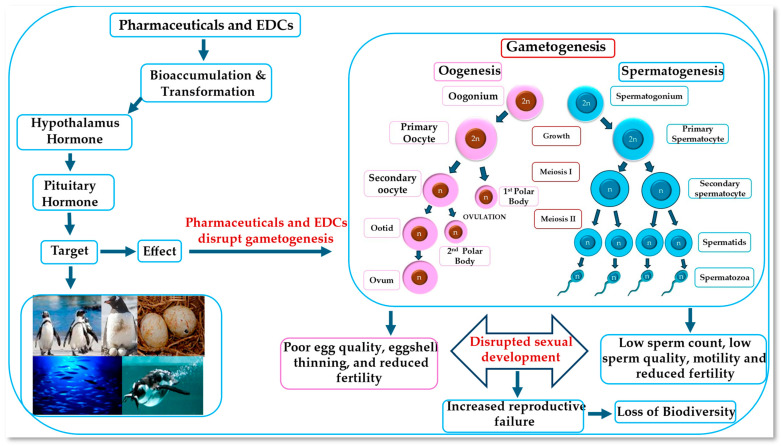
Mechanisms by which EDCs disrupt gametogenesis in the hypothalamic–pituitary–gonadal (HPG) axis.

Pharmaceutics and EDCs can impact virtually all aspects of the reproductive physiology of aquatic fauna, from hormone production at the molecular level to population-level reproductive success. The specific effects depend on the type of pharmaceuticals and EDCs, the concentration, the duration of exposure, the species, and the life stage of the organism. It is well known that pharmaceuticals and EDCs are a growing concern in environmental health [106,107] due to their potential to interfere with the endocrine systems of organisms, including fish, aquatic animals, and mammals. Gametogenesis, the process of producing gametes (sperm and eggs), is a critical step in the reproductive cycle of any animal. It involves cellular events such as cell divisions, germ cell proliferation, and differentiation. These developmental changes lead to the formation of mature gametes, whose role in development is to transfer genomic information to the next generation [82]. The gene expression pattern changes considerably concomitantly with genome remodeling, while genomic information is maintained during this process. Pharmaceuticals and EDCs can mimic or block the action of natural hormones governing gamete formation, leading to various adverse effects on reproduction. These effects include altered sex ratios, reduced fertility [108,109,110], and impaired gamete quality. Pharmaceuticals and EDCs can also affect the development of the reproductive organs, leading to abnormalities such as intersexuality. One of the primary mechanisms by which EDCs disrupt gametogenesis is interference with the hypothalamic–pituitary–gonadal (HPG) axis (Figure 3) [111,112,113]. This complex hormonal system regulates the production and release of sex hormones [114], which are essential for gamete development. These contaminants can disrupt the HPG axis by altering the levels of hormones or by interfering with hormone receptors. For example, certain EDCs can bind directly to estrogen receptors (ERs), mimicking natural estrogens. This receptor-mediated mechanism alters the expression of estrogen-responsive genes and disrupts the balance of endogenous sex hormones, ultimately impairing reproductive development and function. Such direct ER-binding has been observed in various aquatic organisms exposed to EDCs at environmentally relevant concentrations [115]. These disruptions can lead to reduced production of sex hormones, which can impair gametogenesis, ultimately affecting the development and maturation of gametes. For example, some EDCs can interfere with the process of meiosis, which is essential for producing haploid gametes [116,117]. Others can damage DNA, leading to mutations that can impair gamete function. Additionally, pharmaceuticals and EDCs can affect the quality of gametes by altering their morphology or physiology, such as sperm quality, reduced sperm count, and impaired sperm motility, hindering sperm transport and fertilization. Pharmaceutics and EDCs can also affect fertilization success in species with external and internal fertilization by disrupting the synchronization of gamete release, leading to decreased fertilization rates [118]. The specific effects of pharmaceuticals and EDCs on gametogenesis can vary depending on the type of EDC, the exposure level, and the life stage of development, as illustrated in Table 4. Overall, these contaminants pose a significant threat to reproduction and can have long-term consequences for fish, aquatic animals, and mammal populations.

#### 4.1.2. Spawning and Fertilization

As with the impact on gametogenesis, pharmaceuticals and EDCs significantly affect the reproductive processes of aquatic animals, including their spawning behaviors [119,120,121].

They disrupt the complex behaviors associated with spawning, such as the timing of spawning, by interfering with the hormonal signals that regulate reproductive cycles. This can lead to asynchronous spawning, reduced reproductive success, and mismatches between spawning and optimal environmental conditions. Similarly, these contaminants can affect mate choice by altering individuals’ attractiveness or the ability to recognize potential mates [122,123]. Environmental contaminants disrupt mating patterns, reduce genetic diversity, and impair courtship behaviors, including nest building and territorial defense. These disruptions decrease the likelihood of successful mating and fertilization. Furthermore, these environmental contaminants can alter spawning site preferences, leading to suboptimal locations that compromise egg development and larval survival [124,125].

Environmental stressors, including climate change and pollution, pose a significant threat to the early life stages of aquatic oviparous animals. These stages are particularly vulnerable due to their dependence on limited yolk reserves and the need for a rapid transition to exogenous feeding, leading to increased mortality and reduced reproductive success when disrupted. These contaminants can interfere with the endocrine system, leading to various adverse effects, including abnormal development, reduced fertility, and impaired immune function. Studies have shown that exposure to polychlorinated biphenyls (PCBs) can disrupt the reproductive system of fish, leading to intersex conditions and reduced fertility [6,126,127,128]. Furthermore, the transgenerational nature of these effects can have long-lasting consequences for populations and ecosystems. Epigenetic changes induced by environmental contaminants can be passed down to future generations, impacting their health and reproductive capacity.

### 4.2. Impacts of Pharmaceuticals and EDCs on Aquatic Mammal Reproduction

Marine mammals, particularly those at higher trophic levels, exhibit increased sensitivity to persistent pollutants, including pharmaceuticals and EDCs, due to bioaccumulation and biomagnification [129]. Their lipid-rich blubber acts as a significant storage site for lipophilic pollutants, further concentrating these chemicals within their bodies [130,131]. Studies on seals have linked exposure to specific EDCs like PCBs and dichlorodiphenyltrichloroethane (DDT) to adverse reproductive and immune system effects in Baltic populations, contributing to declines [129]. Research on harbor porpoises suggests a correlation between high persistent organic pollutant (POP) burdens and reduced ovarian scar numbers, potentially indicating ovulation inhibition [132]. Furthermore, organochlorine exposure in seals has been shown to disrupt hepatic steroid metabolism and alter sex hormone levels, leading to decreased fertility [133]. Sea lions are also vulnerable to reproductive effects from environmental contaminants, including domoic acid, a marine algal neurotoxin increasingly implicated in reproductive failure, causing spontaneous abortions and premature births. Elevated organochlorine residues have also been associated with a higher incidence of premature births in this species [129]. The ability of domoic acid to cross the placenta indicates a potential for direct harm to developing fetuses [134].

Research on dolphins reveals diverse impacts of pollutants on their reproductive health. Lead and pesticide contamination can disrupt the synthesis of key reproductive hormones, progesterone and testosterone, in Indo-Pacific humpback and bottlenose dolphins [135]. High EDC concentrations in South China Sea dolphin populations raise concerns about reproductive success and potential population declines. PCBs are specifically linked to impaired reproductive processes in dolphins [136], and phthalate exposure in Sarasota Bay dolphins is associated with increased thyroid hormone levels, indirectly affecting reproductive health. An increased prevalence of female reproductive tract pathologies in Northeast Atlantic common dolphins and harbor porpoises suggests a potential link to PCB exposure and endocrine-disrupting effects [137]. Bisphenol A and its analogues are also considered potential reproductive threats to marine mammals, including whales [138].

Bioaccumulation and biomagnification are critical processes in the exposure of aquatic mammals to these pollutants, with higher trophic level species accumulating greater concentrations through contaminated prey [129]. Furthermore, female marine mammals can transfer a significant portion of their pollutant burden to their offspring during gestation and lactation, potentially impacting their development and future reproductive capacity. The enduring impact of legacy pollutants like PCBs and DDT on aquatic mammals’ reproductive health underscores environmental contamination’s long-term consequences, persisting despite use restrictions. The vulnerability to marine toxins like domoic acid highlights the potential for acute reproductive failures due to harmful algal blooms, a risk that may be exacerbated by changing ocean conditions. The increasing detection of emerging pollutants like flame retardants and phthalates suggests continuous threats to reproductive success, necessitating ongoing research and monitoring.

### 4.3. Impacts of Pharmaceuticals and EDCs on Bird Reproduction, with a Focus on Penguins

In avian species, exposure to pharmaceuticals and EDCs can lead to altered sexual differentiation, eggshell thinning, developmental abnormalities, and changes in reproductive behaviors. A well-documented impact is DDE-induced eggshell thinning, which caused widespread reproductive failure in birds of prey [129]. Exposure to planar chlorinated hydrocarbons (PCHs) like dioxins and PCBs is associated with embryo lethality and deformities in birds inhabiting the Great Lakes region, and exogenous estrogen exposure during critical development can induce sex reversal in avian embryos [139].

Regions with penguin colonies, such as Antarctica, research stations, and tourism activities, might contribute to localized pharmaceutical contamination. Given that penguins feed on various marine organisms, there is also a possibility of indirect exposure through the consumption of prey that have accumulated pharmaceutical residues from contaminated waters. The widespread presence of pharmaceuticals in aquatic environments and the potential for penguins to be exposed through multiple pathways underscore the need for further research to directly assess the levels of these contaminants in penguin populations and their habitats. Accordingly, penguin populations in sensitive marine environments are also susceptible to chemical pollution, affecting their reproductive biology [140].

Petroleum-based oils and their by-products may contain or generate EDCs, such as polycyclic aromatic hydrocarbons (PAHs), which are present in crude oil and combustion by-products and are known to interfere with estrogen and androgen pathways. Alkylphenols, another class of EDCs commonly found in oil additives and lubricants, also exhibit estrogenic effects. Moreover, oil spills release a wide range of hydrocarbon compounds into the environment, many of which act as EDCs and disrupt endocrine systems, particularly in aquatic organisms [141]. For example, petroleum contamination negatively impacts hormonal balance and breeding success in Magellanic Penguins (*Spheniscus magellanicus*); even low levels of oil fouling can interfere with reproduction, leading to lower levels of key reproductive hormones and higher corticosterone (a stress hormone) in oiled females, resulting in reduced nesting success. Oil pollution has been shown to induce a significant suppression of key reproductive hormones in *S. magellanicus*, including luteinizing hormone, androgens, and estradiol, which subsequently correlates with reduced nesting success [142].

This evidence suggests a causal link between chemical contamination and impaired reproductive capacity in this avian species. It is well-established in the scientific literature that EDCs can exert negative impacts on reproductive outcomes across a broad spectrum of wildlife species [55], affecting crucial factors such as breeding success rates, circulating endocrine levels, and offspring health. Research in mammalian systems has elucidated the potential for EDCs associated with the ingestion of microplastics to disrupt the hypothalamic–pituitary–gonadal (HPG) axis, a pivotal neuroendocrine axis governing reproductive function [143]. Considering the conserved nature of fundamental endocrine regulatory mechanisms throughout vertebrates, a comparable susceptibility to disruption is plausible in penguins [55]. Consistent with this hypothesis, a 2024 investigation focusing on seabird populations revealed a significant association between microplastic ingestion and the detection of specific EDCs, indicating a potential pathway for reproductive harm in these avian taxa [144]. Moreover, studies examining other anthropogenic disturbances, such as tourism, have shown that they can lead to elevated stress hormone levels and decreased reproductive success in penguins [145], emphasizing the vulnerability of their reproductive physiology to environmental stressors. The potential for emerging contaminants, including nano- and nanoparticles, as well as microplastics, to impact the reproductive success of aquatic fauna at the physiological level has also been reported [146,147]. While direct studies specifically isolating the effects of pharmaceutical exposure on penguin reproduction within the specified timeframe are limited in the provided material, the collective evidence strongly suggests that exposure to EDCs, particularly via microplastic ingestion and associated chemical pollution, can have detrimental consequences for reproductive hormone dynamics and overall reproductive success in penguins.

Per- and polyfluoroalkyl substances (PFAS) and other persistent organic pollutants in penguin populations and eggs are also a growing concern. PFAS have been detected in Adélie penguin eggs in the remote Ross Sea Marine Protected Area, alongside other persistent chemicals like PCBs and flame retardants, highlighting widespread contamination even in seemingly pristine Antarctic ecosystems [148]. Legacy pollutants like PCBs, DDTs, hexachlorobenzene (HCB), endrin, and heptachlor are still present in Antarctic penguin excreta, indicating ongoing exposure [140,149,150].

POPs and trace metals, including mercury, are transported to Antarctica via atmospheric currents and accumulate in penguin tissues, increasing embryo concentrations during later stages of development. Some trace metals, such as cadmium, lead, and mercury, exhibit endocrine-disrupting properties by mimicking natural hormones or interfering with hormone signaling pathways [140,151].

The reproductive physiology of penguins is also influenced by various other environmental stressors, including changes in diet, nutrition, and social stimuli. Contaminants of emerging concern (CECs) detected in nearshore marine environments can negatively impact African penguins’ male fertility and offspring [152]. Chronic exposure to anthropogenic contaminants near Antarctic scientific stations could lead to synergistic or additive effects on various Antarctic marine species, including penguins. Furthermore, climate-related stressors like changes in sea ice cover and ocean acidification could reduce the overall resilience of Antarctic ecosystems, potentially increasing penguin susceptibility to the adverse effects of contaminants. A particularly relevant aspect of EDC pollution for penguins is the role of microplastics. These ubiquitous pollutants in marine environments can act as vectors for EDCs, absorbing and concentrating them on their surfaces, thereby increasing the risk of ingestion by marine organisms, including penguins [147,153]. The sources of EDCs are diverse, ranging from industrial discharges and agricultural runoff to various pharmaceuticals, all of which can lead to the contamination of penguin habitats [154]. The presence of persistent and emerging contaminants in penguins, even in remote regions, underscores the pervasive nature of global pollution and the vulnerability of these seemingly pristine environments. Oil contamination threatens penguin reproductive success by disrupting their hormonal balance and reducing nesting success. While the link between pharmaceutical exposure and reproductive effects in penguins requires further research, its detection is concerning. The interplay of these chemical pollutants with other environmental stressors complicates the challenges faced by penguin populations in maintaining their reproductive health and overall survival.

More importantly, there is a well-established understanding that EDCs can disrupt the endocrine system and negatively impact reproduction in various wildlife species, affecting crucial aspects such as breeding success, hormone levels, and the health of offspring. As stated above, the potential for EDCs carried by ingested microplastics to disrupt the hypothalamic–pituitary–gonadal (HPG) axis, a critical regulator of reproduction, has been demonstrated in mammalian studies [143]. While these findings are in mammals, the fundamental mechanisms of the endocrine system are conserved across vertebrates, suggesting a similar potential for disruption in penguins. A recent study examining seabirds found a link between microplastic ingestion and levels of specific EDCs, indicating a potential pathway for reproductive harm in these avian species [144]. Furthermore, research on other anthropogenic stressors, such as tourism, has shown that they can elevate stress hormones and reduce reproductive success in penguins [145], highlighting the sensitivity of their reproductive system to environmental changes. Although direct studies specifically isolating the impact of pharmaceuticals on penguin reproduction are limited in the literature, the existing evidence strongly suggests that EDC exposure, particularly through microplastics and related chemical pollution, can negatively affect penguin reproductive hormones and overall reproductive success.

### 4.4. Ecological and Population-Level Consequences of Pharmaceuticals and EDCs

Disruptions in reproductive biology at the individual level, caused by pharmaceutical and EDC pollution, can have profound consequences for the long-term health and stability of aquatic populations and the ecosystems they inhabit [155]. Impaired reproduction, manifested as reduced fertility, skewed sex ratios, and developmental abnormalities, can directly lead to declines in population sizes [129]. When these effects occur in keystone species, which play critical roles in maintaining the structure and function of their ecosystems, the resulting population declines can trigger cascading effects throughout the entire food web, leading to broader ecosystem imbalances and a potential loss of biodiversity [156]. The impact of chemical pollution on aquatic mammal and bird populations is further exacerbated by its interplay with other environmental stressors.

Wildlife populations are often simultaneously facing challenges such as climate change, habitat loss, and disease outbreaks. The combined effects of chemical contaminants and these other stressors can be synergistic, meaning their total impact is greater than the sum of their individual effects [157]. For instance, EDCs may interfere with the ability of Arctic marine mammals and seabirds to adequately respond and adapt to the rapid environmental alterations caused by climate change, potentially reducing their resilience to these changing conditions [158]. Additionally, exposure to chemical pollution can weaken the immune systems of aquatic animals, making them more susceptible to the effects of other stressors, such as infectious diseases, further contributing to population declines [159].

The interconnectedness of environmental stressors highlights the complexity of aquatic wildlife’s challenges. Reproductive impairments caused by pollution can reduce a population’s ability to recover from other disturbances, such as extreme weather events or food shortages linked to climate change. The disruption of even a single species through pollution can have ripple effects across trophic levels, affecting predators that rely on the contaminated species as a food source and potentially leading to instability within the entire ecosystem. Therefore, effective conservation strategies must consider the cumulative impacts of multiple stressors, including chemical pollution, to ensure the long-term survival of aquatic mammal and bird populations.

### 4.5. Mitigation Strategies

Addressing the pervasive issue of pharmaceutical and EDC pollution in aquatic environments requires a comprehensive approach that incorporates both preventing these contaminants from entering the environment and developing effective methods for their removal or optimization [62] as illustrated in Table 5.

Upstream strategies focus on reducing the release of pharmaceuticals and EDCs at their source. These include implementing pharmaceutical take-back programs to prevent improper disposal of medications. A promising approach is the redesign of pharmaceuticals to minimize their aquatic toxicity and enhance their biodegradability in the environment, often referred to as “green pharmaceuticals”. Other upstream measures involve increasing the extent to which pharmaceuticals are metabolized within the body, selecting less harmful alternatives when available, initiating new prescriptions at lower dosages, and choosing drugs with lower excretion rates. Concepts such as eco-directed sustainable prescribing (EDSP) and using urine-separating toilets to manage pharmaceutical waste at the household level also represent potential upstream mitigation strategies [160].

Downstream strategies aim to remove pharmaceuticals and EDCs from wastewater and contaminated water bodies. These strategies involve using advanced wastewater treatment technologies that go beyond conventional methods. Effective technologies include adsorption processes, ultrafiltration, advanced oxidation processes (AOPs), and various bioremediation techniques, such as mycoremediation (using fungi) and algae [62]. Nanomaterials are being explored for their potential as highly efficient adsorbents and catalysts in water treatment processes [164]. Membrane filtration processes, including reverse osmosis and ultrafiltration, have shown promise in removing a wide range of organic micropollutants [165]. AOPs, which utilize strong oxidants to break down complex organic molecules, are also being investigated for their effectiveness in eliminating EDCs.

Policy interventions, technological advancements, and public awareness initiatives are crucial to combat pharmaceutical and EDC pollution. Implementing stricter regulations on industrial discharges of these chemicals is essential. Promoting the development and use of safer, non-EDC-containing alternatives in manufacturing and consumer products should be encouraged. In agricultural settings, adopting responsible practices that minimize the use and runoff of pesticides and veterinary pharmaceuticals is necessary [164]. Continued investment in research and development of advanced wastewater treatment technologies capable of efficiently removing these contaminants from water is vital. Enhancing monitoring programs to systematically track the presence and levels of EDCs and pharmaceuticals in various water bodies will provide crucial data for risk assessment and management [161,162,163]. Raising public awareness about the potential dangers of EDCs and pharmaceuticals in the environment and encouraging responsible usage and disposal practices can also significantly reduce pollution levels. For pharmaceuticals specifically, the development and implementation of control measures and eco-pharmacovigilance frameworks are needed to minimize their release into the environment from human populations [152].

Several critical areas require further research to enhance our understanding of the impacts of pharmaceutical and EDC pollution and to develop more effective protection strategies for aquatic species. For beta-blockers, future research should investigate non-cardiac toxicity endpoints in aquatic organisms and develop adverse outcome pathways for cardiac dysfunction [166]. More research is needed on the long-term and chronic effects of exposure to mixtures of pharmaceuticals, as this is the reality in most aquatic environments. Investigating the potential sublethal impacts of pharmaceuticals on growth, reproduction, and survival across multiple species and over multiple generations is also crucial [167]. For antidepressants like fluoxetine, further studies are needed to fully understand their long-term effects on the survival and reproductive success of fish populations [168]. Finally, given the focus of this report, specific research into the potential reproductive effects of pharmaceutical exposure in penguin species is warranted.

A holistic approach that combines preventative measures at the source with advanced treatment technologies, supported by robust policies and increased public awareness, is essential to mitigate the harmful effects of pharmaceutical and EDC pollution on aquatic ecosystems. Continued research into the specific impacts on vulnerable species like penguins will provide the necessary scientific basis for informed decision-making and practical conservation actions.

## 5. Penguins as Sentinel Organisms for Pharmaceutical and EDC Pollution on the Reproductive Biology of Aquatic Fauna

Building on the previously discussed reproductive toxicity of pharmaceuticals and EDCs in aquatic organisms, this section focuses on the unique role of penguins as sentinel species within marine ecosystems. As established in earlier sections, long-lived organisms at higher trophic levels are particularly vulnerable to the bioaccumulation and biomagnification of persistent contaminants. Penguins, flightless seabirds that forage at the top of marine food webs, provide a valuable lens through which to assess the ecological and reproductive consequences of such pollution, especially in remote and sensitive regions of the Southern Hemisphere.

Penguins exhibit considerable promise as sentinel organisms for the escalating environmental challenge of pharmaceutical and EDC contamination in marine ecosystems. Their ecological attributes and the pressing need for robust conservation strategies underscore their potential utility. As apex predators within numerous Southern Hemisphere marine food webs, penguins are prone to bioaccumulation of pollutants through dietary intake, thereby offering valuable insights into the overall ecological health of these environments [169,170,171,172]. The documented declines in populations of various penguin species, including the *Spheniscus demersus* (African Penguin) [152,173,174,175], emphasize the imperative to identify and mitigate all contributing anthropogenic stressors, among which pharmaceutical and EDC pollution represent potentially significant yet frequently understudied factors.

Alterations in penguin physiological condition, reproductive success, or ethological patterns attributable to chemical exposure may serve as an early warning system for analogous impacts on other components of the marine biota and potentially human health via seafood or coastal water contamination. The limited dispersal capabilities and year-round residency within the Southern Hemisphere exhibited by many penguin species render them effective integrators of regional contaminant exposure. Furthermore, their well-documented life history parameters [176,177,178] and the relative accessibility for biological sample collection (e.g., feathers, blood, guano) during terrestrial breeding phases and annual molt facilitates longitudinal monitoring programs. The interspecific diversity observed in foraging ecologies and dietary preferences among penguin taxa provides a unique opportunity to elucidate pollution impacts across disparate trophic levels and geographical regions within the marine realm [179,180,181,182]. The data in Table 6 and Table 7 highlight notable geographic and species-specific exposure of penguins to endocrine-disrupting chemicals (EDCs) and pharmaceuticals, offering insight into environmental contamination across polar and sub-polar regions.

As shown in Table 6, EDCs such as PCBs, DDT-related compounds, PAHs, and HCBs were widely detected in various tissues, muscle, liver, blood, plasma, feathers, and eggs, across species including Magellanic, King, Gentoo, Chinstrap, Emperor, Adélie, Rockhopper, Macaroni, and Little Penguins. Magellanic penguins from Brazil showed the highest levels, particularly of PAHs and β-endosulfan. Although Gentoo and Chinstrap penguins in Antarctica exhibited lower concentrations of PCDD/Fs, PCBs, and PBDEs, their presence demonstrates long-range pollutant transport. HCB, despite its ban, was still found in the plasma of several species, showing its persistent nature, with the highest levels in King penguins from the Crozet Archipelago. As shown in Table 7, limited data exist on pharmaceutical contamination in penguins. However, detections were reported in Gentoo and Adélie penguins, with methylparaben and 4-formyl antipyrine identified in their tissues, indicating emerging pharmaceutical pollution in Antarctic ecosystems.

Despite limited studies quantifying pharmaceutical residues in penguins, the pervasive presence of these contaminants in aquatic environments, originating predominantly from wastewater treatment plant effluents and agricultural runoff, suggests plausible exposure pathways for foraging in littoral zones. Similarly, despite a paucity of direct EDC measurements in penguins in the past few years, the ubiquitous distribution of EDCs, including those associated with microplastic debris, presents a salient concern [147,190,191,192]. Penguins may inadvertently ingest microplastics, which can sorb and concentrate EDCs, or consume prey items that have bioaccumulated these substances [144]. Moreover, the potential for maternal transfer of EDCs to avian embryos via eggs warrants investigation regarding developmental impacts on offspring.

Although direct investigations into the effects of pharmaceutical and EDC pollution on penguin reproductive biology are limited, evidence derived from studies on related chemical contaminants, such as petroleum hydrocarbons, demonstrates the capacity of chemical exposure to disrupt critical reproductive hormones and diminish nesting success. The well-established understanding of the endocrine-disrupting mechanisms of EDCs across diverse vertebrate taxa, including the potential for interference with the hypothalamic–pituitary–gonadal (HPG) axis [111,113,193,194], suggests a comparable vulnerability in penguins (Figure 3). The documented association between microplastic ingestion and elevated EDC levels in other seabird species further supports the potential for reproductive impairment in penguins via this exposure route [195,196].

The utility of penguins as indicator species extends to elucidating the broader ecological ramifications of these pollutants on other aquatic fauna. Given the documented adverse effects of pharmaceuticals and EDCs on the reproductive physiology of various fish and invertebrate taxa, observing reproductive perturbations in penguins could serve as a sentinel for analogous widespread risks within shared marine habitats.

Notwithstanding their considerable potential, current research exhibits certain limitations. The paucity of direct investigations quantifying pharmaceutical residues in penguin tissues and the inherent challenges in isolating the effects of these pollutants from other confounding environmental stressors represent significant methodological hurdles. Analytical limitations in detecting and quantifying the often trace concentrations of a diverse array of pharmaceutical and EDC compounds in environmental matrices and biological tissues, and the need for more comprehensive baseline data from relatively pristine penguin populations, also require attention.

Penguins present a compelling case as sentinel organisms for marine pollution, encompassing the increasing concern regarding pharmaceutical and EDC contamination. While extant research within the 2019–2025 timeframe exhibits certain lacunae, the ecological characteristics of penguins, coupled with the established reproductive toxicity of these pollutants in other wildlife, underscore the exigency for further scientific inquiry. Focused research initiatives are essential to fully realize the potential of penguins as effective sentinels, thereby informing evidence-based conservation strategies for these ecologically significant seabirds and the broader aquatic ecosystems they inhabit.

## 6. Conclusions and Future Directions

The evidence synthesized in this report underscores the significant and detrimental effects of pharmaceutical and endocrine-disrupting chemical pollution on the reproductive biology of aquatic mammals and birds.

Various classes of pharmaceuticals, including hormones, beta-blockers, and antidepressants, along with a wide range of EDCs such as PCBs, DDT, flame retardants, and bisphenol A, have been shown to interfere with reproductive processes through diverse mechanisms. These interferences can lead to a cascade of adverse outcomes at the individual level, including hormonal imbalances, altered sexual development, reduced fertility, and behavioral changes.

Of particular concern are the vulnerabilities of penguin populations to these chemical contaminants. Penguins, especially those in remote regions such as Antarctica, are exposed to a complex mixture of persistent organic pollutants and emerging contaminants, highlighting the global reach of pollution. Oil contamination continues to pose a direct threat to their reproductive success by disrupting hormonal balance and reducing nesting success. While the direct reproductive impacts of pharmaceutical exposure in penguins require further investigation, detecting these compounds in nestlings raises concerns about potential long-term consequences.

The ecological and population-level consequences of these reproductive disruptions are far-reaching. Reduced reproductive success at the individual level can translate to significant declines in population sizes, potentially impacting the stability and biodiversity of entire ecosystems. The interplay between chemical pollution and other environmental stressors, such as climate change and habitat loss, further exacerbates the threats to these vulnerable species.

Addressing this critical environmental issue demands urgent and comprehensive action. It is essential to implement a combination of upstream strategies to prevent the release of pollutants and downstream technologies to remove them from aquatic environments. Policy interventions, technological advancements, and public awareness initiatives must work in concert to mitigate these threats and safeguard the future of aquatic mammals and birds, including the sensitive penguin populations. Continued research into the specific impacts of these pollutants on various species will be crucial for informing conservation efforts and ensuring the long-term health of our planet’s aquatic ecosystems.

Several key gaps in our current understanding need to be addressed through future research to firmly establish the role of penguins as sentinel organisms for pharmaceutical and EDC pollution. A primary focus should be conducting studies that directly measure pharmaceutical residues in various penguin species and investigate their specific effects on reproductive parameters, such as hormone levels, breeding success, and offspring health.

Further research is also needed to quantify EDC levels in penguins across different geographical regions and species, with a particular emphasis on the relationship between EDC accumulation and the ingestion of microplastics.

Investigating the potential combined effects of mixtures of pharmaceuticals and EDCs on penguin reproductive biology is another critical area for future study, as organisms in the wild are often exposed to complex cocktails of pollutants.

Establishing long-term monitoring programs that integrate the measurement of these pollutants in penguin tissues and their environment with detailed assessments of penguin reproductive success and overall health will be crucial for understanding trends and long-term impacts.

Developing and applying sensitive biomarkers to detect early signs of reproductive disruption due to pharmaceutical and EDC exposure would also significantly enhance our monitoring capabilities.

Finally, conducting comparative studies across different penguin species and other relevant aquatic fauna would improve our understanding of the broader ecosystem impacts of these pollutants and the extent to which penguins can serve as indicators for other vulnerable species.

## Figures and Tables

**Table 1:** Occurrence of pharmaceuticals in surface waters.

Location	Chemicals (Levels)	Ref.
Vembe Rivers, South Africa	Caffeine (94 ng/L to 975 ng/L), Nevirapine (7 ng/L to 166 ng/L), Lopinavir (42 ng/L), Acetaminophen (292 ng/L to 427 ng/L), Fluconazole (outside the instrument calibration range, nq), Sulfamethoxazole (nq), Clindamycin (nq), Carbamazepine (21 ng/L)	[12]
Isipingo River, South Africa	Caffeine (ND—3.68 µg/L), sulfamethoxazole (ND—1.28 µg/L), diclofenac (ND—2.44 µg/L), and ibuprofen (ND—1.26 µg/L)	[13]
Rivers of Curitiba in Brazil	Azithromycin (326 to 3340 ng/L), ivermectin (130–3340 ng/L), and hydroxychloroquine (304–3314 ng/L)	[14]
Preto, Turvo and Atibaia Rivers in Brazil	Acetaminophen (157–7449 ng L^−1^), caffeine (60–122,520 ng L^−1^), diclofenac (62–176 ng L^−1^), and sulfathiazole (34–40 ng L^−1^)	[15]
Yangtze River, Wuhan, China	Ibuprofen, caffeine, diclofenac, paracetamol, carbamazepine, propranolol, triclosan	[16]
Rivers across Sichuan, China	Amoxicillin, ampicillin, cephalexin, cefotaxime, enrofloxacin, levofloxacin, norfloxacin, moxifloxacin, sulfadiazine, sulfamethoxazole, oxytetracycline, tetracycline, chlortetracycline, chloramphenicol, clindamycin	[17]
Reshape River, Beijing, China	Metformin hydrochloride, Adamantanamine and Rimantadine hydrochloride (170.9 ng/L), Amantadine (170.75 ng/L), rimantadine hydrochloride (0.15 ng/L), Acyclovir, Penciclovir, Ganciclovir, Chloroquine diphosphate, Lamivudine, Ribavirin, Arbidol hydrochloride, Memantine hydrochloride (0.21 ng/L), Oseltamivir, Moroxydine hydrochloride (30.25 ng/L), Imiquimod	[18]
Kamphuan Stream, Thailand	Diclofenac (249 ng/L), Gemfibrozil (265 ng/L), Metformin (6247 ng/L), Naproxen (103 ng/L), Fexofenadine (5107 ng/L), Gabapentin (3063 ng/L), Ibuprofen (174 ng/L)	[19]
Istanbul Strait, Türkiye	Fluoxetine (4.05 to 69.8 ng/L), Serotonin (1.42 to 32.84 ng/L)	[20]
Buriganga River, Bangladesh	Metronidazole (970 ng/L), Sulfadiazine (790 ng/L), Levofloxacin (710 ng/L), Ciprofloxacin, Amoxicillin, Doxycycline (190 ng/L), Azithromycin (120 ng/L), Lincomycin	[21]
Lempa River Basin, El Salvador	Sulfamethoxazole (23 μg/L)	[22]

**Table 2:** Occurrence of EDCs in surface waters.

Location	Chemicals (Levels)	Ref.
Urban rivers of Southern Mexico	17β-estradiol (1.36 ng/L), estriol (3.11 ng/L), 17α-ethinylestradiol (0.66 ng/L), bisphenol A (30.49 ng/L), 4-nonylphenol (5.95 ng/L), 4-tert-octylphenol (3.58 ng/L)	[23]
Selangor River Basin, Malaysia	Bisphenol A, bisphenol S, bisphenol F, perfluorooctanesulfonate, perfluorooctanoic acid, 17α-ethynylestradiol, and 17β-estradiol	[24]
Yangtze River, Wuhan, China	4-nonylphenol (5.20–49.59 ng/L), Bisphenol-A (<90.44 ng/L)	[16]
Fangchenggang Bay, South China Sea	Androstenedione (3.5 ng/L), Methyltestosterone (3.25 ng/L), Nandrolone (0.05 ng/L), 17α-Hydroxyprogesterone (1.05 ng/L), Norethindrone (0.35 ng/L), Hydrocortisone (19.75 ng/L), Prednisone (5.20 ng/L), Triamcinolone Acetonide (0.45 ng/L), Estriol (5.20 ng/L), Ethynyl Estradiol (3.75 ng/L), Estrone (0.05 ng/L), 17α-Estradiol (0.05 ng/L)	[25]
Rivers of Wuhan, China	4-n-nonylphenol (22–109 ng/L), Octylphenol (25.9–73.7 ng/L), Bisphenol A (93.3–258 ng/L), Bisphenol S (0.42–3.30 ng/L)	[26]
Lempa River Basin, El Salvador	Bisphenol A (2 μg/L)	[22]

**Table 3:** Reproductive modes and strategies in aquatic animals, mammals, and penguins.

Feature	Aquatic Animals (Excluding Mammals and Penguins)	Aquatic Mammals	Penguins
Fertilization	Both internal and external. External fertilization (spawning) is common in many bony fish, amphibians, and marine invertebrates. Internal fertilization occurs in some fish (e.g., sharks, and some bony fish like guppies and Tilapia), crustaceans, and molluscs	Internal.	Internal.
Mode of Development	Oviparity is common, with external development in many species. Viviparity and ovoviviparity occur in some fish and a few amphibians. Parthenogenesis is observed in some aquatic invertebrates.	Viviparity. Embryonic diapause occurs in some pinnipeds.	Oviparity, laying one or two eggs depending on the species.
Parental Care	Ranges from absent (common in broadcast spawners) to elaborate care (nest building, guarding eggs, mouth brooding in some fish like Tilapia).	Extensive maternal care is typical, with prolonged nursing. Cooperative care in some species (e.g., dolphins). Paternal care is generally limited or absent.	Extensive biparental care is common, with both parents involved in incubation and feeding chicks. Chicks often form crèches.
Mating Strategies	Highly diverse, including monogamy in some fish, polygamy in others, and broadcast spawning with no pair bonds in many invertebrates and some fish. Sequential hermaphroditism in some fish.	Vary widely, including monogamy in some smaller cetaceans, polygyny in many pinnipeds and some whales, and promiscuity in others. Competition among males is common.	Most species are monogamous during a breeding season, often with mate fidelity in subsequent years. Serial monogamy in King Penguins.
Key Examples and Specific Strategies	Fish: Wide range (e.g., external fertilization in salmon, internal fertilization in sharks, mouth brooding in Tilapia). Amphibians: Primarily external fertilization (e.g., frogs). Marine Invertebrates: Diverse (e.g., broadcast spawning in corals, budding in sponges, internal fertilization in cephalopods). Micropyle in fish eggs for sperm entry.	Whales: Long migrations, complex songs, intense male competition, single calf with extensive maternal care. Dolphins: Year-round mating, cooperative mating, strong mother-calf bonds. Seals: Seasonal breeding, diverse mating systems, embryonic diapause. Sea Otters: Year-round breeding, polygynous, delayed implantation, maternal care.	Emperor Penguin: Single egg incubated by male on ice. King Penguin: Single egg in a brood pouch, serial monogamy. Adélie Penguin: Stone nests, biparental care.

**Table 4:** Comparison of key reproductive parameters in aquatic fauna.

**Parameter**	**Aquatic Animals (Examples)**	**Aquatic Mammals (Examples)**	**Penguins (Examples)**
Gestation Period/Incubation Period	Bony Fish (General): Days to weeks. Amphibians (General): Days to weeks	Humpback Whale: Gestation: 11–11.5 months; Bottlenose Dolphin: Gestation: 12 months; Harbor Seal: Gestation: ~11 months; Sea Otter: Gestation: 4–12 months	Emperor Penguin: Incubation: ~64 days; King Penguin: 50–60 days. Adélie Penguin: Incubation: ~36 days.
Clutch/Litter Size	Bony Fish (General): Hundreds to millions; Amphibians (General): Hundreds to thousands	Humpback Whale: 1; Bottlenose Dolphin: 1; Harbor Seal: 1; Sea Otter: 1 (rarely 2)	Emperor Penguin: 1. King Penguin: 1. Adélie Penguin: 2.
Age at Sexual Maturity	Bony Fish (General): Months to years; Amphibians (General): Months to years	Humpback Whale: 4–10 years; Bottlenose Dolphin: 5–13 years. Harbor Seal: 3–7 years. Sea Otter: 2–6 years.	Emperor Penguin: 5–6 years. King Penguin: 3–6 years. Adélie Penguin: 3–6 years.
Frequency of Reproduction	Bony Fish (General): Annual or multiple times; Amphibians (General): Annual	Humpback Whale: Every 2–3 years. Bottlenose Dolphin: Every 3–5 years. Harbor Seal: Annual. Sea Otter: ~Annual	Emperor Penguin: Annual. King Penguin: Twice every 3 years. Adélie Penguin: Annual.

**Table 5:** Mitigation strategies for pharmaceutical and EDC pollution in aquatic environments.

Strategy Category	Specific Mitigation Strategy	Brief Description	Ref.
Upstream	Pharmaceutical Take-Back Programs	Collection of unused medications to prevent improper disposal	[160]
Upstream	Green Pharmaceuticals	Designing drugs with lower environmental impact and better biodegradability	[160]
Upstream	Sustainable Prescribing	Optimizing dosage and drug selection to minimize environmental release	[160]
Downstream	Advanced Wastewater Treatment	Technologies like adsorption, oxidation, bioremediation for removing contaminants	[62]
Downstream	Bioremediation	Using biological agents like fungi and algae to degrade pollutants	[62]
Policy/Awareness	Stricter Regulations	Limiting industrial discharges of EDCs and pharmaceuticals	[161,162,163]
Policy/Awareness	Public Education	Raising awareness about responsible use and disposal of medications and EDC-containing products	[161,162,163]

**Table 6:** Occurrence and concentrations of endocrine-disrupting chemicals (EDCs) in penguin species.

Penguin Species	EDC	Concentration	Ref.
Magellanic penguins (*Spheniscus magellanicus*) from the southeastern coast of Brazil	4,4′-Dichlorodiphenyldichloroethylene (DDE)	83 ng/g dry weight in muscle and 160 ng/g dry weight in liver.	[183]
	Dichlorodiphenyltrichloroethane (DDT)-related compounds	27.0 ± 41.4 ng/g dry weight in muscle and 50.3 ± 82.6 ng/g dry weight in liver	
	Hexachlorocyclohexanes (HCH)	7.75 ± 6.30 ng/g dry weight in muscle and 17.9 ± 21.2 ng/g dry weight in liver	
	β-endosulfan	110 ng/g dry weight	
	Endosulfan sulfate	155 ng/g dry weight in muscle	
	Polychlorinated Biphenyls (PCBs)	57.7 ± 95.6 ng/g dry weight in muscle and 133 ± 221 ng/g dry weight in liver	
	Polycyclic Aromatic Hydrocarbons (PAHs)	142 ng/g dry weight in muscle and 1711 ng/g dry weight in liver	
King penguins (*Aptenodytes patagonicus*)	Benzotriazole-based ultraviolet stabilizers (UV-BTs)	0.057 ng/mL in the blood	[184]
	DDE	12.7 ng/mL in the blood	
	PCBs	19.1 ng/mL in the blood	
Gentoo penguins (*Pygoscelis papua*) from the Antarctic Peninsula	perfluorooctanoic acid (PFOA)	0.70 ± 0.28 ng/g dry weight in feathers	[185]
	perfluoro pentanoic acid (PFPeS)	0.84 ± 0.01 ng/g dry weight in feathers	
	sodium dodecafluoro-3H-4,8-dioxa-nonane-1-sulfonate (NaDONA)	0.36 ± 0.11 ng/g dry weight in feathers	
Gentoo penguin from the South Shetland Islands, Antarctica	Polychlorodibenzo-p-dioxins and furans (PCDD/Fs)	3.87 pg/g lipid weight in eggs	[186]
	PCBs	4710 pg/g lipid weight in eggs	
	Polybrominated diphenyl ethers (PBDEs)	123 pg/g lipid weight in eggs	
Chinstrap penguin (*Pygoscelis antarticus*) from South Shetland Islands, Antarctica	Polychlorodibenzo-p-dioxins and furans (PCDD/Fs)	3.89 pg/g lipid weight in eggs	
	PCBs	3200 pg/g lipid weight in eggs	
	Polybrominated diphenyl ethers (PBDEs)	48.8 pg/g lipid weight in eggs	
Gentoo penguin from Crozet archipelago (subantarctic)	Hexachlorobenzene (HCB)	0.12 ± 0.02 µg/g wet weight in plasma	[187]
King penguin from Crozet archipelago (subantarctic)	HCB	0.42 ± 0.29 µg/g wet weight in plasma	
Adélie penguin from Adélie Land, Antarctica	HCB	0.16 ± 0.03 µg/g wet weight in plasma	
Emperor penguin (*Aptenodytes forsteri*) from Adélie Land, Antarctica	HCB	0.22 ± 0.07 µg/g wet weight in plasma	
Southern rockhopper penguin (*Eudyptes chrysocome*) from Crozet archipelago (subantarctic)	HCB	0.14 ± 0.06 µg/g wet weight in plasma	
Macaroni penguin (*Eudyptes chrysolophus*) from Crozet archipelago (subantarctic)	HCB	0.29 ± 0.08 µg/g wet weight in plasma	
Little Penguins (*Eudyptula minor*) from Phillip Island, Victoria, Australia	PCBs	12.9 ± 11.3 ng/g wet weight in blood	[188]
	DDTs	3.5 ± 2.7 ng/g wet weight in blood	

**Table 7:** Occurrence and concentrations of pharmaceuticals in penguin species.

Penguin Species	Pharmaceutical/Drug Metabolite	Concentration	Ref
Gentoo Penguin	Methylparaben	4.46 to 11.6 ng/g wet weight in muscles	[189]
	4-Formyl antipyrine	4.76 to 12 ng/g wet weight in eggs	
Adélie Penguin (*Pygoscelis adeliae*) from West Antarctic Peninsula	4-Formyl antipyrine	4.57 to 6.0 ng/g wet weight in eggs	

## Data Availability

No new data were created or analyzed in this study.

## Abbreviations

AOPs	Advanced oxidation processes
PFAS	Per- and polyfluoroalkyl substances
PBDEs	Polybrominated diphenyl ethers
EDSP	Eco-directed sustainable prescribing
HPG	Hypothalamic–pituitary–gonadal
DDE	Dichlorodiphenyldichloroethylene
EDCs	Endocrine Disrupting Chemicals
POPs	Persistent organic pollutants
TBT	Tributyltin
